# Overtime Work and the Incidence of Long-term Sickness Absence Due to Mental Disorders: A Prospective Cohort Study

**DOI:** 10.2188/jea.JE20200382

**Published:** 2022-06-05

**Authors:** Yosuke Inoue, Shuichiro Yamamoto, Andrew Stickley, Keisuke Kuwahara, Toshiaki Miyamoto, Tohru Nakagawa, Toru Honda, Teppei Imai, Akiko Nishihara, Isamu Kabe, Tetsuya Mizoue, Seitaro Dohi

**Affiliations:** 1 National Center for Global Health and Medicine, Tokyo, Japan; 2 Hitachi, Ltd., Ibaraki, Japan; 3 Department of Preventive Intervention for Psychiatric Disorders, National Institute of Mental Health, National Center of Neurology and Psychiatry, Tokyo, Japan; 4 Teikyo University Graduate School of Public Health, Tokyo, Japan; 5 NIPPON STEEL CORPORATION, East Nippon Works, Chiba, Japan; 6 OH Support, Kanagawa, Japan; 7 Azbil Corporation, Tokyo, Japan; 8 KUBOTA Corporation, Tokyo, Japan; 9 Mitsui Chemicals, Inc., Tokyo, Japan

**Keywords:** workplace, occupational health, healthy workers effect, prospective studies, Asia

## Abstract

**Background:**

Although previous research has focused on the association between long working hours and several mental health outcomes, little is known about the association in relation to mental health-related sickness absence, which is a measure of productive loss. We aimed to investigate the association between overtime work and the incidence of long-term sickness absence (LTSA) due to mental disorders.

**Methods:**

Data came from the Japan Epidemiology Collaboration on Occupational Health Study (J-ECOH). A total of 47,422 subjects were followed-up in the period between April 2012 and March 2017. Information on LTSA was obtained via a study-specific registry. Baseline information was obtained at an annual health checkup in 2011; overtime working hours were categorized into <45; 45–79; 80–99; and ≥100 hours/month.

**Results:**

During a total follow-up period of 211,443 person-years, 536 people took LTSA due to mental disorders. A Cox proportional hazards model showed that compared to those with less than 45 hours/month of overtime work, those with 45–79 hours/month were at a lower risk of LTSA due to mental health problems (hazard ratio [HR] 0.63; 95% confidence interval [CI], 0.56–0.71) while those with overtime work of ≥100 hours/month had a 2.11 (95% CI, 1.12–3.98) times higher risk of LTSA due to mental health problems.

**Conclusion:**

Engaging in excessive overtime work was linked with a higher risk of LTSA due to mental health problems while the lower risk observed among individuals working 45–79 hours/month of overtime work might have been due to a healthy worker effect.

## INTRODUCTION

While many countries have introduced statutory working time limits to protect workers’ health,^1^ working long hours is still commonplace^2^ and has been linked with negative health outcomes.^3^^–^^5^ In particular, mental health conditions have been studied extensively in relation to long working hours in countries across the world. For example, a meta-analysis by Virtanen et al^5^ that aggregated estimates from 28 studies reported that working long hours was associated with depressive symptoms (pooled odds ratio [OR] 1.14; 95% confidence interval [CI], 1.03–1.25) with a stronger association found in Asian countries (pooled OR 1.50; 95% CI, 1.13–2.01). Other mental health outcomes that have been associated with long working hours include anxiety,^6^^,^^7^ psychological stress,^8^ and suicidal thoughts.^9^

Despite this research, several issues remain to be addressed. First, previous studies often relied on self-reported information on mental health outcomes,^10^^–^^12^ which was not medically corroborated and thus subject to measurement error (eg, recall bias, social desirability bias). Second, previous studies on the association between long working hours and mental health outcomes did not focus on severe symptoms such that they caused productivity loss (eg, sickness absence [SA]). Several studies have investigated the association between working hours and SA, but not specifically in relation to mental health problems.^13^^–^^16^ Third, we are not aware of any previous studies that have examined the association between long working hours and mental health outcomes longitudinally while accounting for censored cases. Such cases can bias examined associations, as it is possible that those working long hours are more likely to be lost to follow-up.

To address these research issues the current study will examine the prospective association between long working hours (in terms of overtime work hours) and long-term sickness absence (LTSA) due to mental health problems in Japan using a survival analysis technique. There is reason to believe that it may be especially important to examine the association in Japan. Long working hours are common^17^^,^^18^ with a government survey estimating that Japanese full-time workers undertake an average of 7.7 hours of overtime work per week.^19^ Poor health due to overwork is also frequently observed as reflected in the creation of terms such as “karoshi” (ie, death from overwork) and “karojisatsu” (ie, a suicide death from overwork) that specifically denote this phenomenon.^18^ Despite this, previous research in Japan has produced conflicting findings; for example, while Kato et al^17^ and Suwazono et al^20^ reported a significant association between long working hours and poorer mental health, Uchida and Morita^21^ and Tokuyama et al^22^ did not find any evidence of a significant association.

Against this backdrop, the aim of the current study was to examine if long working hours (hours of overtime worked per month) were associated with the incidence of medically certified LTSA due to mental health conditions among Japanese workers.

## METHODS

### Study design and participants

This study used data from the Japan Epidemiology Collaboration on Occupational Health Study (J-ECOH). J-ECOH is an ongoing epidemiological study investigating the health determinants of Japanese workers.^23^^–^^25^ For the present study, we used the data of three participating companies that provided information on overtime working hours. Of the 64,633 full-time workers who received a health check-up in Fiscal Year 2011 (or 2010, if information was not available in 2011) in these companies, we excluded: those who did not provide information on overtime work (*n* = 7,922), those who were aged <20 years old or ≥60 years old in 2011 (*n* = 4,632); those who were on LTSA in April 2012 (*n* = 125); those with a history of cancer (*n* = 461), cardiovascular disease (CVD) (*n* = 490) or mental illness (*n* = 1,164) as having any of these conditions may be associated with employment restrictions; and those with missing information on any of the covariates (*n* = 105). We further excluded employees without follow-up information (ie, those who did not attend any subsequent health examinations or whose information on mortality, or resignation after LTSA was unavailable) (*n* = 2,312). Consequently, the analytic sample consisted of 47,422 workers (40,180 males and 7,242 females).

The study protocol was approved by the Ethics Committee of the National Center for Global Health and Medicine, Japan (NCGM-G-001140). Informed consent was assumed with not opting-out on the in-company bulletin board with participants having the option of withdrawing their participation at any time during the course of the study. This procedure is in line with the Japanese Ethical Guidelines for Epidemiological Research for observational studies that use existing data.

### Setting

A study-specific registry was created in April 2012 to collect information on LTSA (ie, SA cases that lasted 30 consecutive days or longer^26^), cardiovascular events (ie, myocardial infarction and stroke), and death. While the scope of the available sickness absence data varied among the participating companies, all the companies were able to provide information on sickness absence lasting for 30 consecutive days or longer. Thus, we confined our SA data collection to information on LTSA. Information was also obtained throughout the study period from the annual health checkup, which is mandatory under the Industrial Safety and Health Act. These data were used to follow study participants and determine the type and date of events they experienced. If no such events were recorded, participants were considered to be under observation until the date of the last health checkup. We used information on medical certificates provided by primary doctors; these certificates were submitted by the employees to their companies when applying for paid SA. The International Classification of Diseases 10th Revision (ICD-10) was used to code the diagnoses.

### Outcome

The primary outcome of this study was the onset of LTSA due to all mental and behavioral disorders (F00–F99) during the period between April 1, 2012 and March 31, 2017. We also examined specific secondary outcomes: (1) LTSA due to mood (affective) disorders (ICD-10: F30–39); and (2) LTSA due to neurotic, stress-related and somatoform disorders (F40–F48).

### Explanatory variable

We used self-reported questionnaire information on overtime work, which was collected at each worksite as a categorical variable (ie, the response options provided).^27^^,^^28^ Two companies provided baseline information on overtime work per month while the other company provided information on daily working hours. For the company where data on daily working hours were available, we calculated monthly overtime working hours by subtracting 8 hours from the daily working hours to get overtime work hours per day and then multiplied this figure by 20 days (4 weeks). A conversion table between work hours and overtime working hours is provided in eFigure 1. In this study, we classified monthly overtime into the following four categories: <45 hours, 45–79 hours, 80–99 hours, and ≥100 hours.

### Covariates

Information was obtained on age (in years), sex, smoking status (current smoker; non-current smoker), body mass index (BMI) categories based on measured weight and height (<18.5; 18.5–24.9; 25.0–29.9; ≥30 kg/m^2^), hypertension, diabetes, and dyslipidemia at the baseline. Participants were judged to be hypertensive if their systolic blood pressure was ≥140 mm Hg, diastolic blood pressure was ≥90 mm Hg, or they took antihypertensive medication. Diabetes was defined as a fasting plasma glucose level ≥126 mg/dL, random glucose level ≥200 mg/dL, HbA1c ≥6.5%, or the current use of anti-diabetic medication. Plasma glucose was measured either using the enzymatic or glucose oxidase peroxidative electrode method. HbA1c was measured either using latex agglutination immunoassay, high-performance liquid chromatography, or the enzymatic method, with results converted to the National Glycohemoglobin Standardization Program (NGSP) equivalent value (%), using the formula A1c (%) = 1.02 × A1c (%; based on the Japan Diabetes Society value) + 0.25. Dyslipidemia was defined as triglycerides ≥150 mg/dL, low-density lipoprotein-cholesterol ≥140 mg/dL, high-density lipoprotein-cholesterol <40 mg/dL (men) and <50 mg/dL (women), or taking anti-dyslipidemia medication.

### Statistical analysis

A Cox proportional hazards regression analysis was used to investigate the association between overtime work and medically certified LTSA due to mental and behavioral disorders. Person-time was calculated from March 31, 2012 (ie, 1 day before the beginning of the study period) to the first day of LTSA, date of death, or date of the last participation in the health examination, whichever occurred first. Those who took LTSA due to other causes were censored on the date of the LTSA. The data were checked to confirm that there was no collinearity between the independent variables.

We adjusted for age and sex in model 1 and further adjusted for possible mediators linking overtime work and LTSA due to mental disorders (ie, current smoking status, BMI categories, hypertension, diabetes and dyslipidemia^29^^–^^33^) in model 2. We also accounted for multiple participants at a worksite. To test the robustness of the study findings, we conducted a sensitivity analysis where we adjusted for occupation (engineering; production; administration; and others) and job grade (manager; others) at one worksite where the information on occupation and job grade was available.

All the statistical analyses were conducted using Stata ver. 15.0 (StataCorp, College Station, TX, USA). The proportional hazards assumption was tested using Schoenfeld’s test. We confirmed that the final model (model 2) did not violate the assumption. Results are presented as hazard ratios (HRs) with 95% confidence intervals (CIs). Statistical significance was set at *P* < 0.05 (two-tailed).

## RESULTS

Table 1 shows baseline characteristics of the study participants stratified by the number of hours overtime worked per month. Compared to those employees who worked 45 hours overtime or less, individuals working 100 hours overtime or longer tended to be male and obese.

**Table 1.** Baseline characteristics of the study participants of the Japan Epidemiology Collaboration on Occupational Health (J-ECOH) Study

	Overwork hours/month			

	<45	45–79	80–99	≥100
*N*	35,109	10,372	1,346	595
Age, mean [SD]	41.5 [10.4]	40.5 [9.4]	40.8 [9.0]	41.1 [8.9]
Sex, male, *n* (%)	28,251 (80.5)	10,027 (96.7)	1,323 (98.3)	579 (97.3)
Current smoking, *n* (%)	12,212 (34.8)	4,239 (40.9)	447 (33.2)	196 (32.9)
BMI categories, *n* (%)				
<18.5 kg/m^2^	1,948 (5.6)	408 (3.9)	35 (2.6)	19 (3.2)
18.5–24.9 kg/m^2^	23,708 (67.5)	7,039 (67.9)	932 (69.2)	405 (68.1)
25.0–29.9 kg/m^2^	7,786 (22.2)	2,419 (23.3)	315 (23.4)	136 (22.9)
≥30 kg/m^2^	1,667 (4.8)	506 (4.9)	64 (4.8)	35 (5.9)
Hypertension, *n* (%)	5,609 (16.0)	1,341 (12.9)	152 (11.3)	73 (12.3)
Diabetes, *n* (%)	2,135 (6.1)	556 (5.4)	66 (4.9)	32 (5.4)
Dyslipidemia, *n* (%)	13,750 (39.2)	4,079 (39.3)	515 (38.3)	268 (45.0)

BMI, body mass index; SD, standard deviation.

**Figure 1.**
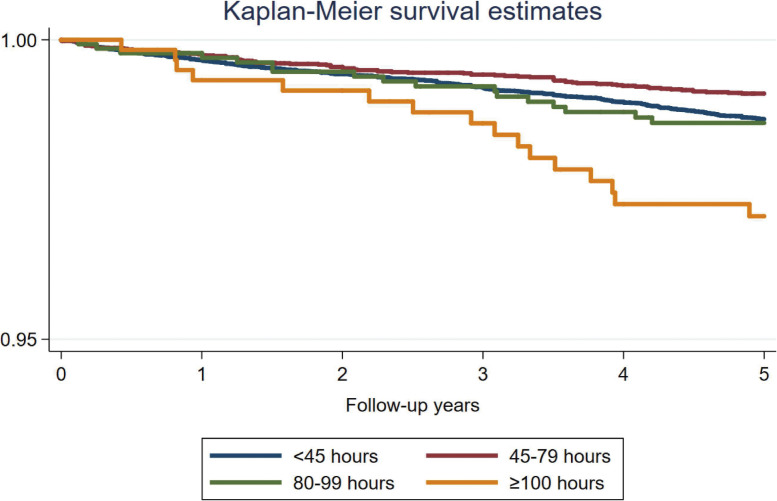
Kaplan-Meier Survival estimates for the association between overtime working hours and long-term sickness absence due to mental health problems

During a total of 211,443 person-years of follow up, there were 536 cases of LTSA due to mental and behavioral disorders (incidence per 1,000 person-years, 2.53; 95% CI, 2.33–2.76). The Kaplan-Meier survival curve is shown in Figure 1 (Log-rank *P* < 0.001). There were 332 cases (61.9%) of LTSA due to mood (affective) disorders and 176 cases (32.8%) of LTSA due to neurotic, stress-related, and somatoform disorders. Table 2 shows the result of a Cox hazards regression model investigating the association between overtime work and the onset of LTSA due to mental disorders. In model 1, compared to those with less than 45 hours of monthly overtime work (reference), those with 45–79 hours of overtime were less likely to take LTSA due to mental and behavioral disorders (HR 0.63; 95% CI, 0.56–0.71). Individuals engaging in 80–99 hours of overtime work did not exhibit an increased risk (HR 0.98; 95% CI, 0.84–1.13), whereas those with ≥100 hours of overtime work had a 2.11 times (95% CI, 1.12–3.98) higher risk of LTSA due to mental and behavioral disorders. These associations did not change materially when the model was further adjusted for possible mediators.

**Table 2.** Adjusted hazard ratios and 95% confidence intervals for medically certified long-term sickness absence due to mental health problems among the Japanese working-age population (2012–2017)

	Overwork hours/month			

	<45	45–79	80–99	≥100
Number of subjects	35,109	10,372	1,346	595
Person-time	155,660	47,006	6,081	2,697
Number of events	417	86	17	16
Crude model HR	1.00 (ref.)	0.68 (0.59, 0.80)	1.04 (0.89, 1.22)	2.22 (1.20, 4.11)
Model 1 HR	1.00 (ref.)	0.63 (0.56, 0.71)	0.98 (0.84, 1.13)	2.11 (1.12, 3.98)
Model 2 HR	1.00 (ref.)	0.63 (0.55, 0.72)	0.99 (0.86, 1.14)	2.11 (1.10, 4.07)

HR: hazard ratio.

Model 1 was adjusted for age (in years) and sex, while model 2 was further adjusted for possible mediators linking working hours and long-term sickness absence due to mental health problems: current smoking (yes/no), body mass index categories (<18.5; 18.5–24.9; 25.0–29.9; ≥30 kg/m^2^), baseline hypertension, diabetes, and dyslipidemia. We treated worksites as clusters in the analysis.

When the analyses focused on affective conditions, those working 45–79 hours of overtime tended to have a lower risk of LTSA due to mood disorders in model 1 (HR 0.71; 95% CI, 0.51–0.99) and those with 100 hours or longer of overtime work were at a higher risk (HR 1.69; 95% CI, 0.85–3.37) while the latter did not reach statistical significance (Table 3). In the analysis investigating the association between overtime work and LTSA due to anxiety disorders, individuals with 45–79 hours of overtime work per month had a lower risk of LTSA (HR 0.49; 95% CI, 0.29–0.81), whereas among those with ≥100 hours of overtime work there was a significantly higher risk of LTSA (HR 2.43; 95% CI, 1.21–4.91).

**Table 3.** Adjusted hazard ratios and 95% confidence intervals for medically certified long-term sickness absence due to mood disorders (F30–F39) or neurotic, stress-related and somatoform disorders (F40–F48) among the Japanese working-age population (2012–2017)

	Overwork hours/month			

	<45	45–79	80–99	≥100
Number of subjects	35,109	10,372	1,346	595
Person-time	155,660	47,006	6,081	2,697
**Mood (affective) disorders (F30**–**F39)**				
Number of events	251	60	13	8
Crude HR model	1.00 (ref.)	0.79 (0.57, 1.11)	1.33 (0.84, 2.11)	1.84 (0.96, 3.54)
Model 1 HR	1.00 (ref.)	0.71 (0.51, 0.99)	1.20 (0.76, 1.89)	1.69 (0.85, 3.37)
Model 2 HR	1.00 (ref.)	0.70 (0.49, 1.00)	1.20 (0.77, 1.87)	1.68 (0.85, 3.31)
**Neurotic, stress-related and somatoform disorders (F40**–**F48)**				
Number of events	144	22	4	6
Crude model HR	1.00 (ref.)	0.51 (0.31, 0.83)	0.71 (0.30, 1.69)	2.41 (1.19, 4.85)
Model 1 HR	1.00 (ref.)	0.49 (0.29, 0.81)	0.70 (0.28, 1.75)	2.43 (1.21, 4.91)
Model 2 HR	1.00 (ref.)	0.49 (0.30, 0.80)	0.72 (0.29, 1.81)	2.47 (1.20, 5.07)

HR: hazard ratio.

Model 1 was adjusted for age (in years) and sex, while model 2 was further adjusted for possible mediators linking working hours and long-term sickness absence due to mental health problems: current smoking (yes/no), body mass index categories (<18.5; 18.5–24.9; 25.0–29.9; ≥30 kg/m^2^), baseline hypertension, and dyslipidemia. Baseline diabetes was not incorporated in the analysis for neurotic, stress-related, and somatoform disorders as it violated the proportional hazard assumption in the analyses for mood disorders. We treated worksites as clusters in the analysis.

In the sensitivity analysis where the study participants were limited to those working at one work site where information on occupation and job grade were available, similar findings were produced to those reported in the main analysis. More specifically, those working 45–79 hours overtime were at a lower risk of LTSA even after adjusting for occupation and job grade while the elevated risk of LTSA due to mental disorders among those working for 100 hours or longer became non-significant possibly due to the small sample size (eTable 1).

## DISCUSSION

This study investigated the association between overtime work and medically certified LTSA due to mental and behavioral disorders among workers in Japan. Using data from 47,422 workers with 211,443 person-years of follow up, we found a U-shaped association between overtime working hours and LTSA due to mental health problems. Specifically, compared to those working <45 hours of overtime per month, individuals working ≥100 hours were at a higher risk of LTSA due to mental and behavioral disorders (HR 2.11), while an inverse association was observed among those with 45–79 hours of overtime work per month (HR 0.63). The association seemed more pronounced for LTSA due to anxiety than affective disorders.

Our study finding of a higher risk of LTSA due to mental disorders among those with ≥100 hours/month of overtime work accords with the result of a recent systematic review and meta-analysis, which aggregated estimates from 28 studies from 35 countries, that showed that long working hours were associated with a higher risk of depressive symptoms (OR 1.14).^5^ While previous studies have used several different cut-off values to define “long” working hours, one common definition has been 55 working hours/week which has been found to increase the risk of mental health outcomes.^5^ Assuming an employee who works 5 days a week and 4 weeks a month with 40 hours statutory work (eFigure 1), then working 55 hours/week is equivalent to 60 hours/month of overtime work. Thus, it is not unexpected that we detected an increased risk of LTSA among those who engaged in excessive overtime work (ie, ≥100 hours/month). The risk of LTSA due to mental disorders among those with 80–99 hours of overtime work per month, which was above the common definition of long working hours used in previous studies on depression, was not elevated. Although it is uncertain why this interval was not associated with an increased risk for LTSA due to mental disorders, this result highlights the utility of examining a range of overtime intervals as well as the need for future research to use both quantitative and qualitative methods to better understand the association between excessive overtime work and physical and mental health outcomes.

Several mechanisms might underlie the association between long working hours and mental health outcomes. First, previous studies have reported an inverse association between working hours and sleep duration^34^^–^^36^; cumulative fatigue associated with insufficient sleep can be a risk factor for mental health outcomes. Second, it is also possible that circadian misalignment due to an irregular sleeping pattern between weekdays and weekends might increase the risk of mental health outcomes.^37^ Third, psychological stress associated with long working hours may have an indirect effect on mental health outcomes via the adoption of unhealthy stress coping behaviors. For example, some research has indicated that those who work long hours may have a higher risk of unhealthy alcohol consumption,^38^ while other research has linked alcohol consumption with depressive symptoms.^39^

It is also of note that the magnitude of the association between overtime working hours (ie, ≥100 hours/month) and LTSA tended to be larger when anxiety rather than affective disorders was used as the outcome. Kleppa et al^40^ also showed that the association between overtime work (defined as working 49–100 hours/week) and mental health outcomes was slightly stronger for anxiety (OR 1.67; 95% CI, 1.36–2.06) than depressive symptoms (OR 1.50; 95% CI, 1.17–1.93) among male workers in Norway. While it is uncertain what underlies this difference and whether it is meaningful in clinical terms, it is possible that it might result from the nature of anxiety disorders themselves, manifesting as a more immediate and direct reaction to severe stress, and/or as an adjustment disorder from failing to adapt to a stressful environment (such as overtime working hours in the context of the current study).

The lower risk of LTSA due to mental disorders observed among those with 45–79 hours of overtime work seems to contradict the findings reported by Virtanen et al.^5^ However, it accords with a series of studies that showed an inverse association between long working hours and all-cause SA; a systematic review by Bernstrøm and Houkes^14^ concluded that there is moderately strong evidence that long working hours (defined as working more than 48–50 hours a week) were inversely associated with SA. One plausible interpretation for the lower risk of LTSA due to mental disorders associated with 45–79 hours of overtime is that it is due to a healthy worker effect^14^^,^^41^ (ie, healthy workers work longer hours than unhealthy workers). It has also been argued that those who are motivated to work longer may be less likely to take SA.^14^ In addition, although we followed-up participants prospectively, some unmeasured differences between those who worked long hours and those who did not (eg, work engagement^42^) might have caused this counterintuitive phenomenon. Selecting those with less than 45 hours of overtime work as a reference group might also have been problematic in terms of introducing bias as it is possible that being in this category might itself be a marker of employment restrictions on working hours due to poorer health.

This study has several limitations that should be mentioned. First, we only had information on overtime work in each specific month, so we were unable to determine the extent to which the exposure had lasted for individual workers (ie, potential misclassification of working time). Second, information on baseline overtime work was self-reported and thus subject to reporting bias, although the validity of self-reported working hours has previously been shown to be high among a subsample of the J-ECOH study.^43^ Third, information on working hours was collected independently by representatives at each worksite, and because of this we were not able to distinguish between those who did not engage in any overtime work and those with >0 to <45 hours of overtime work (ie, introducing possible measurement error). Fourth, our study participants might not have been representative of the working population in Japan (especially the female participants, given their low proportion). For example, we collected data from large companies, which had a relatively generous employment protection system. If no such protection was available, those with severe mental disorders might have become unemployed or retired instead of taking LTSA. It is also possible that those working for large companies might have a better socioeconomic status than the general population. Fifth, we did not collect some potentially important information at baseline, which would have helped us to better interpret the findings (eg, marital status, work environment, educational level, chronic health conditions, social support, or a history of mental disorder at one worksite). For example, health issues might have prevented people from engaging in overtime work or they might have been instructed not to do so by an occupational physician. Sixth, some confounding factors may have also existed that we were not able to examine, such as debt leading both to long working hours and stress or job characteristics that increase both working hours and the risk for worse mental health (eg, low job control, lack of autonomy, and repetitive work).

In terms of the practical implications of our findings, it should be noted that several political commitments have been made to improve occupational health in Japan since the baseline information on overtime working hours was collected in 2011. For example, the Stress Check Program^44^ was introduced in 2015, whereby all workplaces with 50 or more employees should conduct a questionnaire survey at least once a year to identify those at risk of mental health problems. In addition, labor reform laws were enacted in 2018 in order to change the way people work; the new legislation limits overtime work to (1) 720 hours per year; (2) 100 hours in a month; and (3) a monthly average of 80 hours for 2 to 6 consecutive months.^45^ Despite this, as yet, it is uncertain whether these policies have been effective in terms of protecting and improving employees’ health. A focus is now needed on determining whether excessive working hours have been reduced in the wake of these legislative changes.

### Conclusion

In an occupational cohort of Japanese employees, compared to less than 45 hours of monthly overtime work, 45–79 hours of overtime work per month was associated with a lower risk of medically certified LTSA, while overtime work equal to or in excess of 100 hours per month was associated with a higher risk of LTSA. The lower risk among those who work for an extra 45–79 hours per month might have been due to a healthy worker effect.

## APPENDIX A. SUPPLEMENTARY DATA

eFigure 1. Conversion chart for overtime work hours and working hours for the Japan Epidemiology Collaboration on Occupational Health Study (2012–2017)

eTable 1. Adjusted hazard ratios and 95% confidence intervals for medically certified long-term sickness absence due to mental health problems with additional adjustment for job grade among workers at one company (2012–2017) (N = 39,459)
