# Supplementary material for: Overtime Work and the Incidence of Long-term Sickness Absence Due to Mental Disorders: A Prospective Cohort Study
Source: J Epidemiol. 2022 Jun 5;32(6):283–9. doi: 10.2188/jea.JE20200382 (PMC9086305; doi:10.2188/jea.JE20200382)
Supplement: Supplementary file 1 [file je-32-283-s001.pdf]

eFigure 1. Conversion chart for overtime work hours and working hours for the Japan Epidemiology Collaboration on Occupational Health Study (2012–2017)

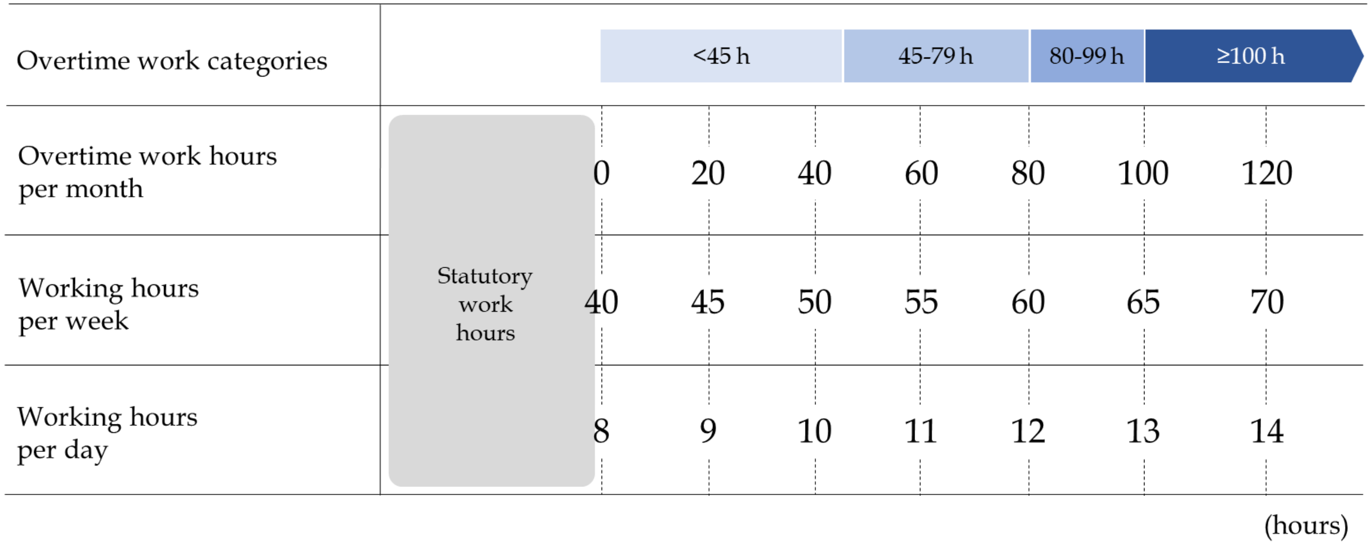

This figure assumes an employee works 5 days a week and 4 weeks a month.

eTable 1. Adjusted hazard ratios and 95% confidence intervals for medically certified long-term sickness absence due to mental health problems with additional adjustment for job grade among workers at one company (2012–2017) (N = 39,459)

	Overwork hours/month			
	<45	45–79	80–99	≥100
Number of subjects	27,874	10,017	1,089	479
Person-time	122,366	45,379	4,985	2,203
Number of events	283	78	13	9
Crude model HR	1.00 (ref.)	0.74 (0.58, 0.96)	1.13 (0.65, 1.97)	1.77 (0.91, 3.44)
Model 1 HR	1.00 (ref.)	0.67 (0.52, 0.86)	0.98 (0.56, 1.72)	1.60 (0.82, 3.13)
Model 2 HR	1.00 (ref.)	0.67 (0.52, 0.86)	0.99 (0.57, 1.73)	1.57 (0.80, 3.06)

HR, hazard ratio.

Model 1 was adjusted for age (in years), sex, occupation and job grade while model 2 was further adjusted for possible mediators linking working hours and long-term sickness absence due to mental health problems, i.e., current smoking (yes/no), body mass index categories (<18.5; 18.5–24.9; 25.0–29.9; ≥30 kg/m^2), baseline hypertension, diabetes, and dyslipidemia. We treated worksites as clusters in the analysis.
